# Ostéonécrose de la tête fémorale compliquant un vissage préventif d’une épiphysiolyse fémorale supérieure: cas clinique et revue de la littérature

**DOI:** 10.11604/pamj.2020.37.112.23228

**Published:** 2020-10-02

**Authors:** Alberic Lionel Kolontchang Gatchou, Mario Sanguina, Penance Agbelele, Alexandre Manga, Abdoul Kikar, Nourredine Ameur, Pierre Girard, Bachar Zerkly, Farikou Ibrahima

**Affiliations:** 1 Sorbonne Université, Paris, France,; 2 Service de Chirurgie Orthopédique et Traumatologique, Groupe Hospitalier Publique Sud de l’Oise, Creil, France,; 3 Département de Chirurgie Orthopédique et Traumatologique, Université de Abomey Calavi, Cotonou, Benin,; 4 Service de Chirurgie Orthopédique et Traumatologique, Centre National de Réhabilitation des Personnes Handicapées, Yaoundé, Cameroun,; 5 Service de Chirurgie Orthopédique et Traumatologique, Centre Hospitalier d’Amiens Picardie, Amiens, France,; 6 Département de Chirurgie Orthopédique et Traumatologique, Faculté de Médecine de l’Université de Yaoundé I, Yaoundé, Cameroun

**Keywords:** Épiphysiolyse, vissage, préventif, ostéonécrose, Epiphysiolysis, screwing, preventive, osteonecrosis

## Abstract

L’attitude thérapeutique à adopter sur la hanche radiologiquement «normale» d’un enfant présentant une épiphysiolyse fémorale supérieure sur la hanche controlatérale, reste controversée. Nous rapportons le cas d’un jeune adolescent de 12 ans qui a développé une ostéonécrose de la tête fémorale radiologiquement «normale» et asymptomatique, sur la hanche gauche qui a été fixée de façon prophylactique dans le cadre d’une épiphysiolyse fémorale aigue survenue sur la hanche controlatérale. Huit mois après la première chirurgie, le patient a développé des signes d’ostéonécrose avasculaire de la tête fémorale. Des critères spécifiques existent pour permettre de poser l’indication d’un vissage préventif ou d’une simple surveillance rapprochée. Malgré ces critères le risque de survenue d’ostéonécrose sur la hanche «saine», n’est pas nul.

## Abstract (en)

The therapeutic approach for the treatment of children with radiologically “normal” hip presenting with epiphysiolysis of the contralateral upper femur is controversial. We here report the case of a 12-year-old boy with osteonecrosis of radiologically “normal” and asymptomatic left femoral head prophylactically fixed due to acute femoral epiphysiolysis of the contralateral hip. Eight months after first surgery, the patient developed symptoms of avascular osteonecrosis of the femoral head. Specific criteria to be used in evaluating the most effective treatment between preventive screw or simple close monitoring exist. Despite these criteria, the risk of osteonecrosis in the “healthy” hip is not zero.

## Introduction

L’ostéonécrose de la tête fémorale est une complication de l’épiphysiolyse ou d’un traitement inadapté occasionnant une interruption de la vascularisation de type terminale [1]. Ainsi, toute réduction intempestive au bloc opératoire par des manœuvres forcées est pourvoyeuse d’ostéonécrose par arrachement ou étirement vasculaire [2]. Les enfants atteints d’épiphysiolyse unilatérale ont un risque de développer un glissement controlatéral ultérieur de la tête fémorale de 20% à 80% [3]. Ainsi, le traitement prophylactique de la hanche controlatérale «normale» doit être considéré, même s’il est controversé [4] et doit tenir compte du risque de complication d’une chirurgie supplémentaire corrélé avec le risque d’épiphysiolyse controlatérale ultérieure et d’arthrose secondaire. Certains facteurs de risque d’épiphysiolyse fémorale supérieure (EFS) plus usuelles [5] ont été décrits dans nombreuses publications: le jeune âge, l’obésité, l’insuffisance rénale, des anomalies endocriniennes (hypothyroïdie, déficit en hormone de croissance), et l’origine ethnique.

D’autres facteurs de risques très importants sont également mis en cause dans la survenue d’EFS; il s’agit des forces mécaniques anormales agissant à travers le cartilage de croissance de la tête fémorale [6]. La rétroversion relative ou réelle du col fémoral, l’orientation physaire de la tête fémorale, les changements de la force physaire, l’asphéricité tête-col fémorale, ont toutes été impliquées comme causes mécaniques potentielles d’EFS [5]. Des facteurs radiographiques prédictifs du risque d’épiphysiolyse controlatérale, ont été identifiés. Ils permettent une plus grande précision dans les indications de fixation prophylactique dans la hanche controlatérale; il s’agit de: l’angle de Southwick, l’angle de la pente physaire postérieure (postérior sloping angle), le score d’Oxford modifié, l’angle alpha [5]. Le cas clinique que nous vous présentons, décrit une complication grave due à la fixation prophylactique percutanée de la hanche d’un adolescent ayant eu une EFS unilatérale. Le patient et ses tuteurs légaux ont été informés que le cas devait être soumis à publication, et un consentement éclairé a été obtenu.

## Patient et observation

**Figure 1:**
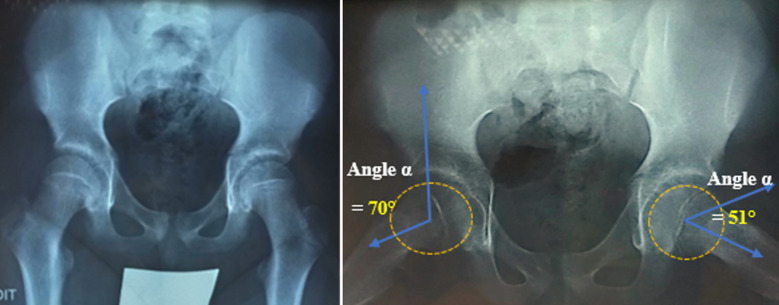
radiographies de face et de profil des hanches montrant un déplacement de la tête fémorale droite

Un jeune adolescent de 12 ans avait été emmené en consultation pour un rendez-vous post opératoire, présentant une douleur de la hanche gauche évoluant depuis 3 mois et une légère boiterie du même côté. Huit mois plutôt, il avait été emmené aux urgences pour douleur de la hanche droite évoluant depuis trois jours. L’examen à l’entrée avait révélé un indice de masse corporelle de 28kg/m^2^ (poids 80,6kg; taille, 170cm); un signe de Drehmann positif (rotation externe passive et automatique de la hanche lors de sa flexion), une absence de limitation de la mobilité des hanches, pas d’altération des signes généraux, pas d’impotence fonctionnelle, état hémodynamique stable. La biologie était normale. Le bilan paraclinique avait permis de noter à la radiographie une épiphysiolyse fémorale supérieure aigue droite de type II (angle de Southwick = 32°). Sur la hanche controlatérale «saine», l’angle de Southwick était de 15°, l’angle d’inclinaison postérieure était de 14,5°, le score d’Oxford modifié était de 19 sur 48, l’angle alpha était de 51° (Figure 1). Après un bilan préopératoire et une consultation préanesthésique, les différentes options thérapeutiques ont été présentées au patient et à la famille, qui ont accepté le traitement chirurgical prophylactique de la hanche controlatérale (gauche) «normale» initialement.

**Figure 2:**
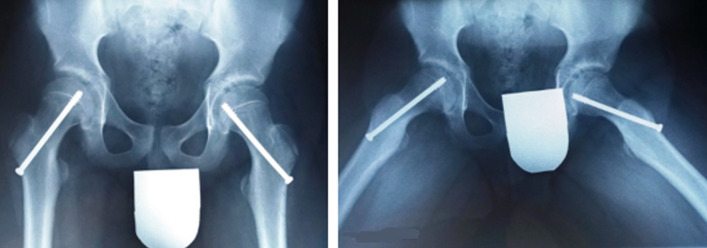
incidence radiographique de face et de profil des deux hanches à J1 post opératoire, confirmant la position du matériel d’ostéosynthèse dans les deux hanches

La chirurgie a été réalisée avec le patient positionné sur une table orthopédique, sans exercer de traction, car cette dernière est associée à des risques de nécrose [6, 7]. La procédure opératoire a été réalisée par voie percutanée avec l’utilisation d’un amplificateur de brillance. Sur les deux fémurs, une broche filetée de 2,5mm a été insérée dans le col sous scopie, puis nous avons utilisé un moteur électrique avec mèche canulée de 5mm, sur un guide-mèche, puis le vissage a été réalisé par des vis canulées de 7,3mm de diamètre de part et d’autre. Au fémur droit, sur l’incidence de face la vis était située au 1/3 inferieur du col et sur l’incidence de profil située au centre du col; sur le fémur gauche, en incidence de face la vis était située au 1/3 inférieur du col et sur l’incidence de profil, la vis était orientée de façon postéro-antérieure. Les radiographies de contrôle obtenues en post opératoire immédiat, après la chirurgie ont confirmé les positions du matériel d’ostéosynthèse (Figure 2). En post opératoire précoce, l’appui a été autorisé à gauche, mais pas à droite. Les suites opératoires étaient simples à 1 mois.

**Figure 3:**
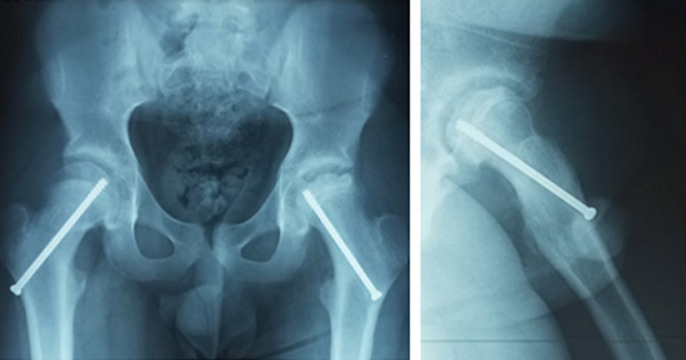
huit mois post opératoire, sur l’incidence de face et de profil on note une asymétrie de la forme de la tête fémorale gauche, qui est plus petite que la tête fémorale droite, un élargissement de l’interligne articulaire; c’est l’ostéonécrose de la tête fémorale à gauche

**Figure 4:**
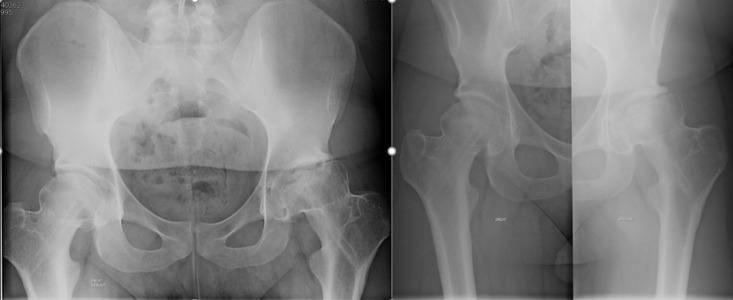
radiographie de contrôle à 9 ans post opératoire initiale, on note la coxarthrose gauche évoluée

**Figure 5:**
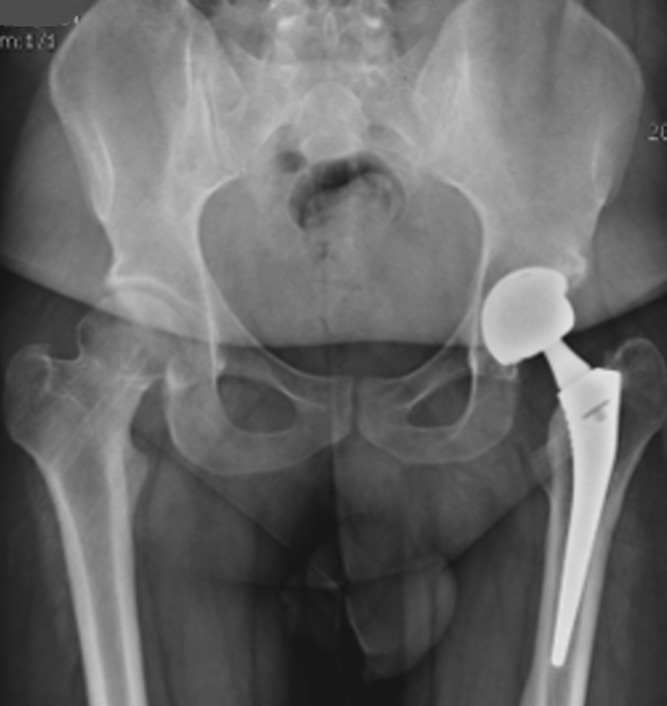
prothèse totale de hanche gauche

À 8 mois, le patient est revenu au rendez-vous, avec une boiterie, et une douleur de la hanche gauche. A la radiographie du bassin, il a été noté une ostéonécrose de la tête fémorale gauche (Figure 3). Le patient a bénéficié d’une ablation du matériel d’ostéosynthèse 3 mois plus tard, donc à 11 mois post opératoire. A 2 ans post opératoire, le patient présentait uniquement une boiterie mais avec absence de douleur à la hanche gauche; il a été également noté un raccourcissement du membre inférieur gauche de 1,5cm avec une limitation de la rotation externe et de la rotation interne de la hanche gauche respectivement à 20° et à 10°. Le patient a été régulièrement suivi les sept années suivantes, il a été dispensé de toute activité sportive, la limitation des mouvements de la hanche gauche s’altérait lentement et progressivement, mais sans douleur. Neuf ans plus tard, après la première chirurgie, il a présenté en plus de son impotence fonctionnelle, une douleur de la hanche gauche, et a évolué vers une coxarthrose (Figure 4). L’indication de prothèse totale de hanche a été posée. Une prothèse totale de hanche de type non cimentée avec un couple de frottement céramique-céramique, à double mobilité, a été posée (Figure 5). Deux ans après, le patient ne présente aucune de douleur, il marche de façon autonome, et présente de très bonnes amplitudes articulaires de la hanche gauche. Le score fonctionnel de PMA est à 18.

## Discussion

Les enfants atteints d’épiphysiolyse unilatérale ont un risque de développer un glissement controlatéral ultérieur de la tête fémorale de 20% à 80% [4, 8]. La méthode de fixation idéale est controversée à cause des risques de bilatéralité passée inaperçue, de glissement ultérieur controlatéral, de survenue d’éventuelles complications du traitement chirurgical. Et donc les risques doivent être mesurés devant chaque cas d’EFS [3]. Le score d’âge osseux d’Oxford modifié (Tableau 1) est le meilleur indicateur du risque que les patients présentant une EFS unilatérale développent une EFS controlatérale [4]. Les patients avec des scores d’Oxford de 16, 17 et 18 ont une probabilité de 96% de développer un EFS controlatérale [8, 9]. Le score d’Oxford modifié de notre patient était de 19. Le PSA (posterior sloping angle) de notre patient était de 14,5°. Le vissage prophylactique a été recommandé pour les patients ayant un angle d’inclinaison postérieur supérieur à 12° [10]. L’angle de Southwick de notre patient était de 15°. Son augmentation est corrélée à la sévérité du glissement dans l’EFS. L’angle alpha fournit une mesure quantitative du degré d’asphéricité de la tête fémorale. Les patients avec des angles alpha significativement élevés (> 50,5°) sont plus à risque d’EFS controlatéral et doivent bénéficier d’une surveillance plus approfondie ou d’une fixation prophylactique de la hanche [5]. L’angle alpha de notre patient était de 51°.

**Tableau 1:** score d'Oxford modifié

Physe	Points	Description
Tête Fémorale	5	Col droit, pas de fovéa, bec médial d'épiphyse
	6	Epiphyse plus large que le col, fovéa bien définie
	7	Début de fermeture de la physe
	4	Simple point d'ossification
Grand trochanter	5	Connexion régulière entre le point d'ossification et le col
	6	Début de fermeture de la physe
Petit trochanter	3	Physe clairement ouverte
	4	Physe partiellement fermée
	5	Physe fermée
Cartilage triradié	1	Physe clairement ouverte
	2	Physe partiellement fermée
	3	Physe fermée
Ilium	3	Absence d'ossification apophysaire
	4	Présence d'ossification apophysaire

L’ensemble de ces valeurs montrent que notre patient avait des signes de pré-glissement de l’épiphyse fémorale supérieur controlatérale. Ce qui a justifié, en accord avec le patient et la famille le vissage prophylactique de la hanche «saine», qui 8 mois plus tard évoluera vers l’ostéonécrose de la tête fémorale. A notre connaissance, c’est la 4^e^ fois que cette pathologie est décrite dans la littérature. Trois auteurs ont déjà rapporté la survenue de cette complication peu ordinaire. Il s’agit de Sankar *et al*. [4] en 2013 aux Etats-Unis dans le Colorado qui fut le premier auteur à la décrire, il a rapporté 2 cas d’ostéonécrose de la tête fémorale de la hanche saine sur une série de 99 patients ayant bénéficié d’un vissage prophylactique sur épiphysiolyse fémorale aigue unilatérale. Ensuite Kroin *et al*. [9] en 2015 aux Etats-Unis à Chicago, a fait état de 2 cas cliniques d’ostéonécrose aseptique de la tête fémorale 8 mois après vissage prophylactique et enfin Chargui *et al*. [8] en 2018 en Suisse à Genève qui a publié un cas clinique d’un en enfant qui a développé une ostéonécrose aseptique de la tête fémorale 6 mois après vissage prophylactique.

Plusieurs hypothèses peuvent expliquer la survenue de cette complication; la vascularisation du col et de l’épiphyse proximale du fémur a pu être compromise; quoique nous n’avons effectué ni de traction ni de rotation excessive de la hanche, les vaisseaux réticulées de l’épiphyse ont pu être lésé lors du positionnement de la broche guide filetée qui était postérolatérale dans l’épiphyse au lieu d’être centrée tel que recommandé [8], certains auteurs introduisent cette broche guide sous amplificateur de brillance à double incidence simultanée de face et de profil et après injection de produit de contraste pour opacifier les vaisseaux [8]; l’augmentation de la température osseuse lors du méchage a pu augmenter le degré de chaleur et donc la destruction irréversible des cellules osseuses. Quoi que nous ayons respecté le point d’entrée de la vis dans le col qui doit être antérieur ou latéral (en cas de glissement minime) tel que recommandé [8], sa position dans l’épiphyse a pu jouer un rôle néfaste sur la vascularisation de l’épiphyse proximale car a été orientée en zone antéro postérieure au lieu d’être centrée [8].

Au vu de l’ensemble de ces facteurs mis en cause, nous recommandons: l’utilisation d’un amplificateur de brillance de génération récente montrant simultanément l’incidence de face et de profil, et avec possibilité d’injection de produit de contraste pour opacifier les vaisseaux épiphysaires, le bon positionnement de la vis au centre de la tête tant sur l’incidence de face que sur l’incidence de profil, l’utilisation d’un moteur pour méchage à 2 phases qui permet de diminuer la chaleur locale et d’éviter l’ostéonécrose thermique, l’utilisation d’un liquide refroidissement comme le sérum physiologique lors du méchage dans le but également de diminuer l’augmentation de la température osseuse.

## Conclusion

La complication grave de notre patient qui a été causée par une fixation préventive, ravive la controverse sur le traitement prophylactique de la hanche controlatérale «normale», et soulève la question de la pertinence du traitement chirurgical dans la prophylaxie du glissement controlatéral. Même si la fixation avec une seule vis canulée est considérée comme une méthode sûre, la décision du choix du traitement prophylactique doit tenir compte des facteurs prédictifs du risque de bilatéralité du glissement et surtout tenir compte des complications éventuelles d’une chirurgie supplémentaire. Par conséquent, le risque d’ostéonécrose de la hanche normale fixée prophylactiquement doit toujours être pris en compte lors de la proposition d’un traitement chirurgical préventif. Il est donc nécessaire lors de la chirurgie prophylactique, de mettre tout en œuvre pour diminuer ce risque d’ostéonécrose de la tête fémorale.
